# Plasma lipidic fingerprint associated with type 2 diabetes in patients with coronary heart disease: CORDIOPREV study

**DOI:** 10.1186/s12933-023-01933-1

**Published:** 2023-08-03

**Authors:** Alejandro Villasanta-Gonzalez, Marina Mora-Ortiz, Juan F. Alcala-Diaz, Lorenzo Rivas-Garcia, Jose D. Torres-Peña, Asuncion Lopez-Bascon, Monica Calderon-Santiago, Antonio P. Arenas-Larriva, Feliciano Priego‑Capote, Maria M. Malagon, Fabian Eichelmann, Pablo Perez-Martinez, Javier Delgado-Lista, Matthias B. Schulze, Antonio Camargo, Jose Lopez-Miranda

**Affiliations:** 1 Lipids and Atherosclerosis Unit, Department of Internal Medicine, Reina Sofia University Hospital, University of Cordoba, Cordoba, Spain; 2 Department of Medical and Surgical Sciences, University of Cordoba, Cordoba, Spain; 3 Instituto Maimonides de Investigación Biomédica de Córdoba (IMIBIC), Córdoba, Spain; 4 CIBER Fisiopatología de la Obesidad y Nutrición (CIBEROBN), Instituto de Salud Carlos III, Madrid, Spain; 5 Department of Analytical Chemistry and Nanochemistry University Institute, University of Cordoba, Cordoba, Spain; 6 CIBER de Fragilidad y Envejecimiento Saludable (CIBERFES), Instituto de Salud Carlos III, Madrid, Spain; 7 Department of Cell Biology, Physiology and Immunology, University of Cordoba, Cordoba, Spain; 8 German Center for Diabetes Research, Munich-Neuherberg, Germany; 9 Department of Molecular Epidemiology, German Institute of Human Nutrition Potsdam-Rehbrücke, Nuthetal, Germany; 10 Germany Institute of Nutrition Science, University of Potsdam, Nuthetal, Germany

**Keywords:** LC–MS, Random survival forest, Lipidomic risk score, Cox

## Abstract

**Objective:**

We aimed to identify a lipidic profile associated with type 2 diabetes mellitus (T2DM) development in coronary heart disease (CHD) patients, to provide a new, highly sensitive model which could be used in clinical practice to identify patients at T2DM risk.

**Methods:**

This study considered the 462 patients of the CORDIOPREV study (CHD patients) who were not diabetic at the beginning of the intervention. In total, 107 of them developed T2DM after a median follow-up of 60 months. They were diagnosed using the American Diabetes Association criteria. A novel lipidomic methodology employing liquid chromatography (LC) separation followed by HESI, and detection by mass spectrometry (MS) was used to annotate the lipids at the isomer level. The patients were then classified into a Training and a Validation Set (60–40). Next, a Random Survival Forest (RSF) was carried out to detect the lipidic isomers with the lowest prediction error, these lipids were then used to build a Lipidomic Risk (LR) score which was evaluated through a Cox. Finally, a production model combining the clinical variables of interest, and the lipidic species was carried out.

**Results:**

LC-tandem MS annotated 440 lipid species. From those, the RSF identified 15 lipid species with the lowest prediction error. These lipids were combined in an LR score which showed association with the development of T2DM. The LR hazard ratio per unit standard deviation was 2.87 and 1.43, in the Training and Validation Set respectively. Likewise, patients with higher LR Score values had lower insulin sensitivity (*P* = 0.006) and higher liver insulin resistance (*P* = 0.005). The receiver operating characteristic (ROC) curve obtained by combining clinical variables and the selected lipidic isomers using a generalised lineal model had an area under the curve (AUC) of 81.3%.

**Conclusion:**

Our study showed the potential of comprehensive lipidomic analysis in identifying patients at risk of developing T2DM. In addition, the lipid species combined with clinical variables provided a new, highly sensitive model which can be used in clinical practice to identify patients at T2DM risk. Moreover, these results also indicate that we need to look closely at isomers to understand the role of this specific compound in T2DM development.

*Trials registration* NCT00924937.

**Supplementary Information:**

The online version contains supplementary material available at 10.1186/s12933-023-01933-1.

## Background

Diabetes mellitus, a metabolic disorder defined by high blood glucose levels (i.e. hyperglycaemia) [1, 2], currently affects 422 million people worldwide according to WHO. T2DM (or non-insulin-dependent diabetes) represents circa 90% of all those cases. Furthermore, the prevalence of T2DM is expected to grow to 643 million patients by 2040 [3], which could have a detrimental impact on public health systems.

The simultaneity of T2DM with CHD raises the risk of mortality by up to 80% compared to the ratio observed across individuals without CHD [4], thus worsening the prognosis for these patients. Faced with the current scenario, there is an urgent need to improve our knowledge about the underlying mechanisms of this disease to find out new strategies to diagnose and treat these patients. Despite T2DM being associated with higher levels of circulating free fatty acids and triacylglycerols, the knowledge of lipid species associated with T2DM remains unclear [5].

Dyslipidaemia associated with T2DM is characterized by increased concentrations of low-density lipoproteins (LDL) cholesterol particles, low levels of high-density lipoproteins (HDL) cholesterol, and high plasma triglycerides [6, 7]. However, this definition of T2DM dyslipidaemia could be seen as inaccurate given the number of different classes observed in multiple molecular species among the lipoproteins and triglycerides-rich particles. A previous study assessing diabetes risk identified two main plasma lipid profiles formed by different lipid classes associated with T2DM development [8]. In this study, the risk of T2DM was associated with high levels of triacylglycerols (TGs), diacylglycerols (DAGs), and phosphatidylethanolamines (PEs), and low levels of lysophosphatidylcholines (PCs), lysophosphatidylethanolamines (LPCs), phosphatidylcholine-plasmalogens (PC-PLs), sphingomyelins (SMs), and cholesterol esters (CEs), showing that the profile linked with T2DM is defined by different lipid classes. Despite incipient scientific interest in the lipidomics of T2DM, the literature is still very limited, and further research is required to confirm the role of these species. However, the studies published so far do not distinguish between the different lipidic species and/or isomer pairs within the same lipid family, which could result in inconsistencies between different publications.

In this study, we carried out a highly-sensitive lipidomic protocol capable of defining the compounds at such a level of detail [9]. We aimed to identify which lipid species at baseline were associated with T2DM development in CORDIOPREV, a 7-year dietary interventional study with patients with CHD designed to aid the early detection of patients at risk of becoming diabetics. The identification of lipidic species with predictive power, in combination with clinical variables, may also contribute to explaining which underlying metabolic mechanisms may be linked with T2DM.

## Methods

### Study subjects

The current work was conducted within the framework of the Coronary Diet Intervention with Olive Oil and Cardiovascular Prevention Study (CORDIOPREV; Clinical trials.gov. Identifier: NCT00924937). This is an ongoing prospective, randomized, open, controlled trial with 1002 patients. The patients received conventional treatment for CHD and had their last coronary event took place over 6 months before joining the study. The volunteers followed one of two different dietary models, a Mediterranean or a low-fat diet, for 7 years, in addition to their conventional treatment for coronary heart disease [10].

The patients were recruited principally at the Reina Sofia University Hospital (Cordoba, Spain), with contributions from other health centres in Cordoba and Jaen, between November 2009 and February 2012. The eligibility criteria, design, and methods of the CORDIOPREV clinical trial were already reported [10]. Briefly, patients were eligible if they were i) aged between 20 and 75 years, ii) had established CHD without clinical events in the last 6 months, iii) were willing to follow a long-term dietary intervention and iv) did not have severe diseases, v) did not have an estimated life expectancy of fewer than 5 years. All the patients gave written informed consent to participate in the study. The trial protocol was approved by the ethic committee of Reina Sofia University Hospital in Cordoba (No. 1496/27/03/2009), following the Helsinki Declaration and good clinical practices. The experimental protocol conformed to international ethical standards.

Our study included 462 patients from the CORDIOPREV study (N = 1002). These patients had not been diagnosed with T2DM at the beginning of the study according to specifications from the American Diabetes Association (ADA) T2DM diagnosis criteria [11]. Of these 462 patients, 4 were excluded from the study due to technical difficulties in the analytical procedure, resulting in a final n of 458 patients.

These patients were followed up for a median of 60 months and 107 developed T2DM (incident-DIAB), according to the ADA T2DM criteria [11], by which the incidence of T2DM was evaluated every year as follows: fasting plasma glucose ≥ 126 mg/dL and 2 h plasma glucose in the 75 g oral glucose tolerance test (OGTT) ≥ 200 mg/dL and/or HbA1c plasma levels ≥ 6.5%. The baseline medication of the subjects in the study are shown in Additional file 1: Table S1.

### Study experimental design

The study design has been previously described [10, 12]. In brief, the participants were randomized to receive two diets: an MED diet or an LF diet. The LF diet consisted of < 30% total fat (< 10% saturated fat, 12–14% monounsaturated fatty acids (MUFA) fat, and 6–8% poly-unsaturated fatty acids (PUFA) fat), 15% protein, and a minimum of 55% carbohydrates. The MED diet consisted of a minimum of 35% of calories as fat (22% MUFA fat, 6% PUFA fat, and < 10% saturated fat), 15% protein, and a maximum of 50% carbohydrates. In both diets, the cholesterol content was adjusted to < 300 mg/dL. At the beginning of the study and every year, each patient had a face-to-face interview with a nutritionist to fill in a previously validated 137-item semi-quantitative food frequency questionnaire [13] and a validated 14-item questionnaire to estimate the adherence of the patient to the Mediterranean diet. This questionnaire was then used to produce a Mediterranean diet score [14]. An OGTT was carried out as previously described [15].

### Lipidomic analysis

The protocol to carry out the lipidomic analysis is described elsewhere [9]. Briefly, the analysis was performed using LC separation followed by HESI in negative or positive mode and detection by MS/ MS. The separation was carried out using a Kinetex C18 100 A column (100 mm × 3 mm i.d., 2.6 μm particle size) from Phenomenex (Madrid, Spain) protected with a C18 pre-column (4 mm × 3 mm), also from Phenomenex. The composition of mobile phase A was 60:40 (v/v) deionized water:acetonitrile, while phase B was 85:10:5 (v/v) isopropanol:acetonitrile:deionized water. Both phases contained 5 mM ammonium formate and 0.1% (v/v) formic acid as ionization agents [16].

### Random survival forest

The lipidomic data were normalized using log transformation and scaled in multiples of 1 standard deviation (SD). We then performed a random classification of patients into two different datasets: a Training Set with 274 patients (60% of the total), in which the variables were selected using RSF [17], and a Validation Set, with 184 patients (40% of the total), to validate the results. The lipids with the highest predictive power for T2DM development were identified in the training set using RSF in combination with a backward selection procedure in the training set [18]. This procedure identified 15 lipids out of the 440 from the dataset as closely correlated with T2DM development.

### Lipidomic Risk score building

We performed a Cox proportional hazards regression analysis with the 15 lipids selected by RSF in the Training set to determine the potential use of these lipids as an independent predictor of T2DM development. Next, a LR Score was built by multiplying the coefficients obtained for every lipid in the previous step (the Cox analysis) by its plasma concentration. This LR Score was built into both the Training and the Validation set. Furthermore, patients were classified according to the score generated to carry out a second Cox proportional hazards regression with each one of those variables, adjusted by diet, age, gender, body mass index (BMI), HDL, TGs, and statin intensity treatment. Finally, the predictive capacity of this score was evaluated by classifying the same population with different cut-off points.

### Statistical analysis

We used RStudio [https://cran.r-project.org/, R version 3.6.2 (2019-12-12)] and SPSS statistical software (IBM SPSS Statistics version 21.0) for the statistical analysis of the data. The normal distribution of variables was assessed using the Kolmogorov–Smirnov test. The results are reported with the mean ± standard error of the mean (SEM) for continuous variables and with frequencies for categorical variables. *P*-values ≤ 0.05 were considered statistically significant. The statistical differences in the metabolic variables between groups were evaluated by one-way analysis of variance (ANOVA), and qualitative variables were compared using the Chi-square test. A repeated-measures ANOVA test was used to determine the statistical differences between indexes during the OGTT at baseline and after five years of follow-up. The post hoc statistical analysis was followed by Bonferroni's multiple comparison tests. A generalised lineal model was carried out combining the 15 lipidic isomers and the clinical variables of interest [19, 20].

## Results

### Baseline characteristics of the participants

The baseline characteristics of the subjects in the study are shown in Table 1. The values of BMI, weight, waist circumference, TGs plasma levels, HbA1c, fasting glucose, fasting insulin, and HOMA-IR were higher in the incident-DIAB group than in the non-DIAB group (*p* < *0.05*). Conversely, the insulin sensitivity index (ISI), insulinogenic index (IGI), and disposition index (DI) values were lower in the incident-DIAB group than in the non-diabetic patients at baseline who did not develop T2DM after the follow-up period (non-DIAB) group (*p* < *0.05*). The characteristics of the subjects in the study after a median follow-up of 60 months are shown in Additional file 2: Table S2.

**Table 1:** Baseline characteristics of the population for type 2 diabetes mellitus incidence study

	Incident-DIAB	Non-DIAB	P-value
n	107	355	n/a
Men/women (n)	87/20	302/53	n/a
Age (years)	58.75 ± 0.87	57.33 ± 0.50	0.171
Weight (kg)	85.70 ± 1.47	82.49 ± 0.72	0.037
BMI (kg/m^2^)	31.39 ± 0.47	29.88 ± 0.22	0.002
Waist circumference (cm)	105.28 ± 1.08	101.73 ± 0.57	0.003
Serum triacylglycerols (mg/dL)	132.60 ± 6.60	119.45 ± 3.24	0.059
Total cholesterol (mg/dL)	164.97 ± 3.41	160.65 ± 1.62	0.217
HDL-cholesterol (mg/dL)	43.52 ± 1.04	44.58 ± 0.53	0.355
LDL-cholesterol (mg/dL)	93.40 ± 2.66	91.10 ± 1.33	0.421
CRP (mg/L)	2.88 ± 0.29	2.51 ± 0.17	0.329
HbA1c (%)	6.03 ± 0.03	5.86 ± 0.02	< 0.001
HbA1c (mmol/mol)	42.37 ± 0.36	40.51 ± 0.19	< 0.001
Fasting glucose (mg/dL)	96.18 ± 1.04	92.59 ± 0.53	0.002
Fasting insulin (mU/L)	10.51 ± 0.66	8.34 ± 0.31	0.001
ISI	3.35 ± 0.20	4.32 ± 0.14	0.001
HOMA-IR	3.37 ± 0.30	2.58 ± 0.09	0.001
IGI	0.64 ± 0.30	1.08 ± 0.06	0.025
Disposition Index	0.83 ± 0.05	1.03 ± 0.03	0.003

Means values ± S.E.M

Incident-DIAB: non-diabetic patients at baseline who developed T2DM after a median follow-up of 60 months; non-DIAB: non-diabetic patients at baseline who did not develop T2DM after a median follow-up of 60 months; BMI: body mass index; HbA1c: glycated hemoglobin A1c; ISI: insulin sensitivity index; IGI: insulinogenic index

One-way ANOVA P-values

### Random survival forest

**Fig. 1:**
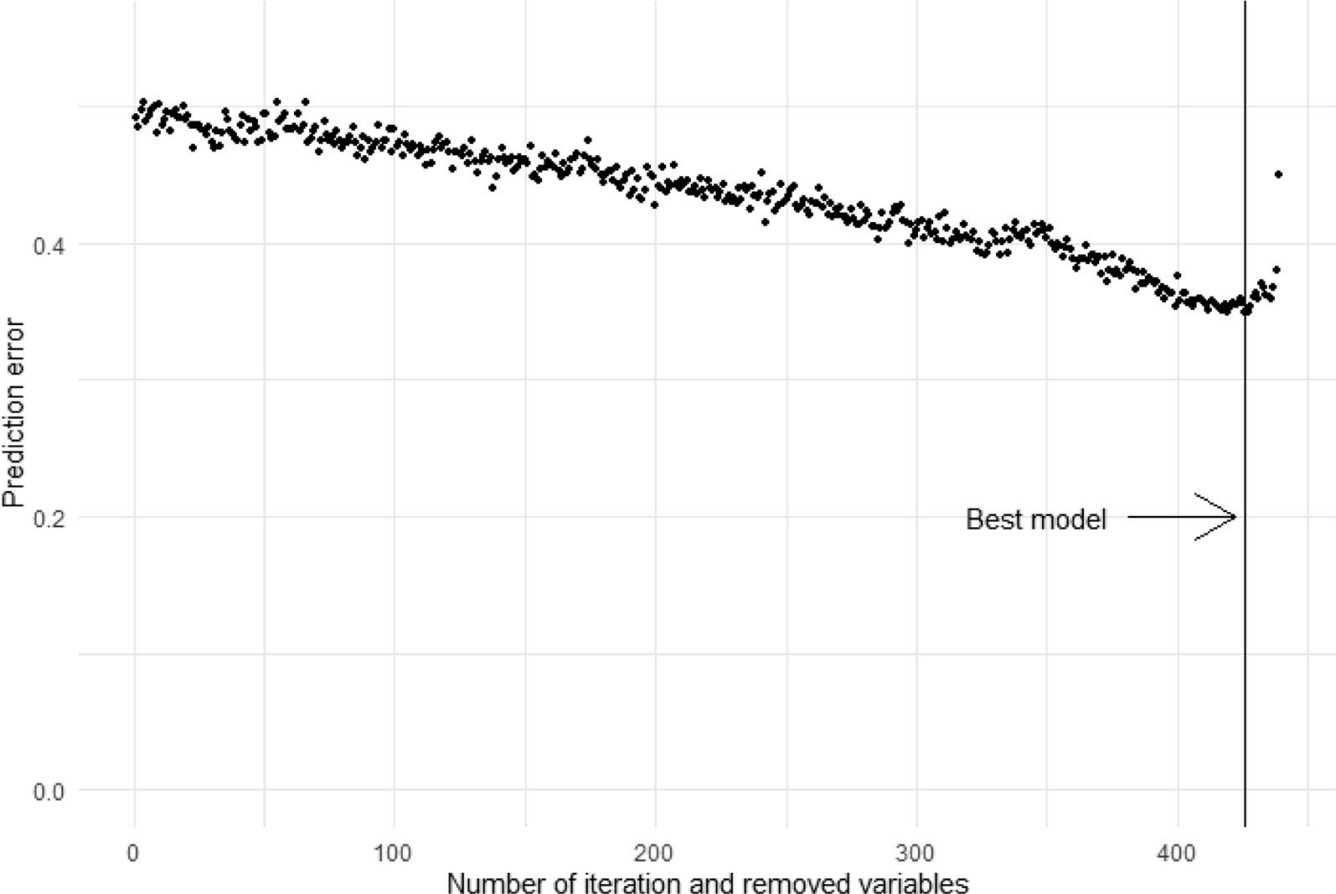
Selection of the best model by Random Survival Forest. Selection in the Training set of lipid species with a higher predictive power for type 2 diabetes, by applying an Random Survival Forest in combination with a backward selection procedure

A stepwise RSF was carried out in the Training Set (60% of patients) to select the lipids with greater predictive power for T2DM development. Thus, the 15 lipids that produced the lowest prediction error, out of a total of 440 lipids variables originally tested, were included in the final model (Fig. 1 and Table 2).

**Table 2:** Selection of lipids included in the model with the lowest prediction error in the Training Set

Lipid	Iteration	Error
PE(16:1/18:1)	426	0.349
PG(16:1/18:1)	427	0.350
PI(18:0/18:1)	428	0.354
PE(16:0/18:1)	429	0.361
PI(18:0/18:2)	430	0.363
PC(P-16:0/18:1)	431	0.360
TG(16:0/16:0/15:0)	432	0.370
TG(16:0/15:0/14:0)	433	0.368
PI(16:0/16:0)	434	0.362
LPC(20:1)	435	0.360
PE(18:0/18:2)	436	0.360
PE(O-20:0/18:0)	437	0.367
PC(P-16:1/18:0)	438	0.380
PS(16:0/18:0)	439	0.450
PG(16:2/20:4)	440	

PE: phosphatidylethanolamine; PG: phosphatidylglycerol; PI, phosphatidyl inositol; PC: phosphatidylcholine; TG: triacylglycerols; LPC: lysophosphatidylcholine; PS: phosphatidylserine

To evaluate the relationship of these lipids with the development of diabetes, an individual Cox proportional regression model was made for each of the 15 selected lipids. In total, 8 of the 15 lipids were directly associated with T2DM development, while 7 of them were inversely associated with T2DM development. It is important to note that different members of the PC, PE, phosphatidyl glycerol (PG), and phosphatidyl inositol (PI) families were associated with both the development and non-development of T2DM (Table 3).

**Table 3:** Association between lipids selected and type 2 diabetes mellitus development in the Training Set, per standard deviation increase

	Coeff	HR	95% CI for HR		Linear Trend
			Lower	Upper	
Model 1					
LPC(20:1)	− 0.43	0.65	0.49	0.88	0.004
PC(P-16:0/18:1)	− 0.20	0.82	0.59	1.14	0.233
PC(P-16:1/18:0)	0.19	1.22	0.88	1.69	0.243
PE(16:0/18:1)	0.00	1.00	0.73	1.38	0.993
PE(16:1/18:1)	− 0.03	0.97	0.71	1.32	0.853
PE(18:0/18:2)	− 0.27	0.76	0.56	1.02	0.072
PE(O-20:0/18:0)	0.16	1.17	0.93	1.47	0.191
PG(16:1/18:1)	0.21	1.23	0.90	1.68	0.194
PG(16:2/20:4)	− 0.09	0.92	0.66	1.28	0.603
PI(16:0/16:0)	− 0.04	0.96	0.74	1.24	0.738
PI(18:0/18:1)	0.14	1.15	0.90	1.46	0.256
PI(18:0/18:2)	− 0.23	0.79	0.59	1.06	0.118
PS(16:0/18:0)	0.48	1.62	0.86	3.07	0.136
TG(16:0/15:0/14:0)	0.18	1.19	0.66	2.14	0.556
TG(16:0/16:0/15:0)	0.12	1.13	0.85	1.51	0.405
Model 2					
LPC(20:1)	− 0.40	0.67	0.49	0.92	0.014
PC(P-16:0/18:1)	− 0.20	0.82	0.57	1.17	0.277
PC(P-16:1/18:0)	0.20	1.23	0.88	1.72	0.223
PE(16:0/18:1)	0.12	1.13	0.80	1.60	0.491
PE(16:1/18:1)	− 0.15	0.87	0.64	1.19	0.391
PE(18:0/18:2)	− 0.33	0.72	0.56	0.93	0.012
PE(O-20:0/18:0)	0.07	1.07	0.84	1.37	0.588
PG(16:1/18:1)	0.25	1.28	0.91	1.80	0.151
PG(16:2/20:4)	− 0.07	0.93	0.65	1.34	0.702
PI(16:0/16:0)	− 0.04	0.96	0.72	1.29	0.799
PI(18:0/18:1)	0.11	1.12	0.86	1.46	0.395
PI(18:0/18:2)	− 0.34	0.71	0.52	0.98	0.035
PS(16:0/18:0)	0.65	1.92	0.95	3.88	0.069
TG(16:0/15:0/14:0)	0.01	1.01	0.54	1.88	0.976
TG(16:0/16:0/15:0)	0.21	1.24	0.91	1.68	0.176

Model 1 was unadjusted and Model 2 was adjusted by age, gender, diet, body mass index, high density lipoproteins-cholesterol, plasma triacylglycerols, and statin intensity treatment

HR: hazard ratio; CI: confidence interval. LPC: lysophosphatidylcholine; PC: phosphatidylcholine; PE: phosphatidylethanolamine; PG: phosphatidylglycerol; PI, phosphatidyl inositol; PS: phosphatidylserine; TG: triacylglycerols

**Fig. 2:**
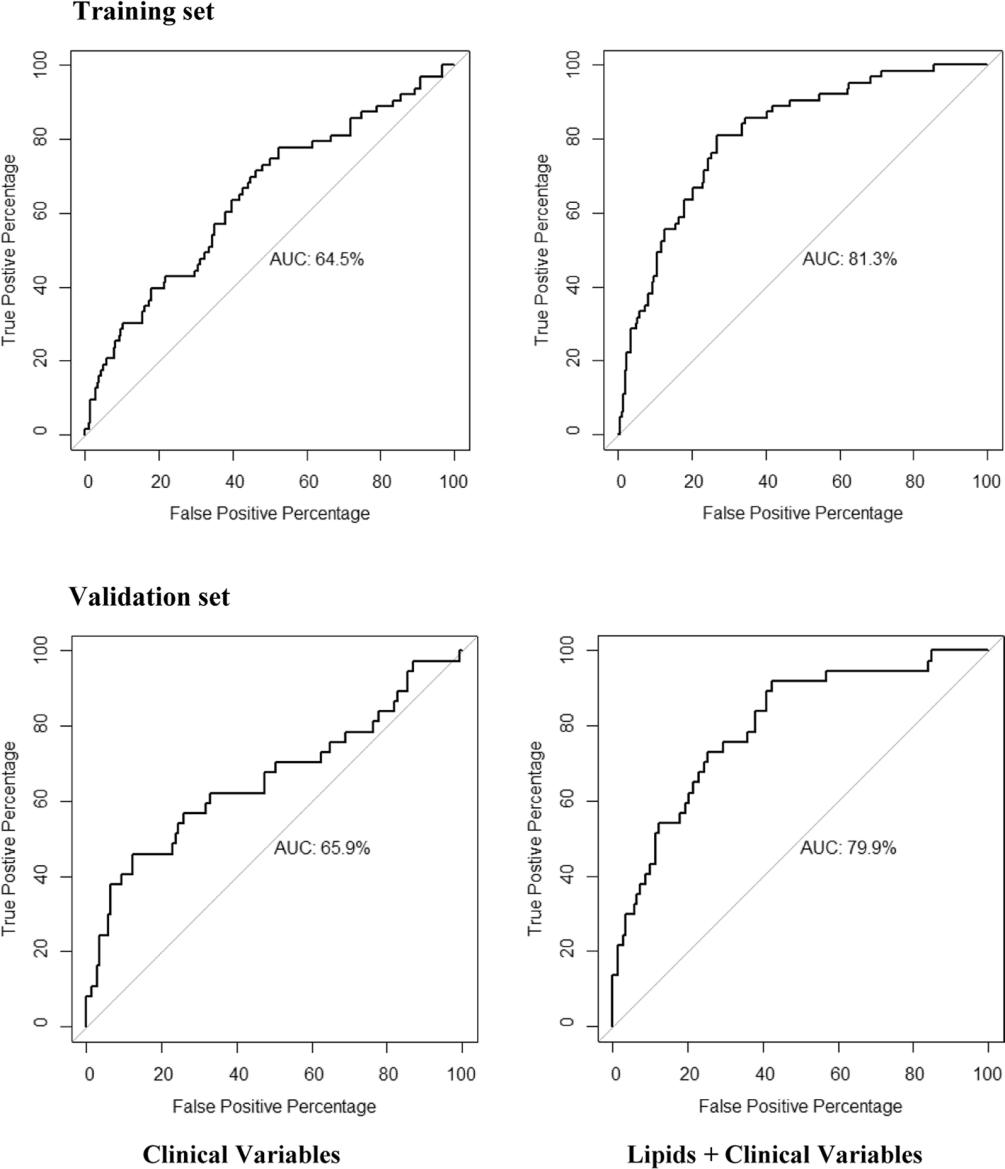
Receiver operating characteristic curves of the model including the clinical variables separately and the model including the 15 lipids and the clinical variables. The clinical variables included: age, gender, diet, body mass index, high density lipoproteins-cholesterol, plasma triacylglycerols, and statin intensity treatment. AUC: area under the curve

Moreover, the model with the 15 lipids produced a C Index of 0.714; but when the clinical variables (i.e., diet, age, gender, BMI, HDL, TGs, and statin intensity treatment) were added into the model, the C Index increased to 0.757, while the clinical variables, taken separately, showed a C Index of only 0.618 (Table 4). In addition, ROC curves of clinical variables separately yielded an AUC of only 0.645, whereas the 15 selected lipids yielded an AUC of 0.771, which rose to 0.813 when the clinical variables were included. Meanwhile, the clinical variables separately yielded an AUC of only 0.645 (Fig. 2A). This difference between the two models, including only clinical variables, and clinical variables plus lipids, was statistically significant according to the DeLong test (*p-value* = 3.16e−06).

**Table 4:** Parameters of the different models

	Training set		Validation set	
	*C-Index*	*ROC*	*C-Index*	*ROC*
Lipids	0.714	0.772	0.703	0.742
Clinical parameters	0.618	0.645	0.653	0.659
Lipids + clinical parameters	0.757	0.813	0.755	0.800

We then tested the predictive power of the RSF model with the selected lipids in the Validation Set. The C Index was 0.703 for lipids, which rose to 0.755 when the clinical parameters were included, which had previously yielded 0.653 when they were tested separately. ROC curves of the selected lipids yielded an AUC of 0.742, which increased to 0.799 when the clinical variables were included, while the clinical variables taken separately yielded an AUC of only 0.659 (Fig. 2B). The DeLong test comparing both models carried out in the Validation Set was also statistically significant (*p-value* = 0.01).

### Results from the score based on the lipidomic profile

A LR Score was built to assess the relationship between the lipidomic profile and T2DM development (see Materials and Methods). To achieve this, the coefficients obtained for each of the 15 lipids in the Cox proportional hazards regression were multiplied by the lipid concentrations in plasma for each subject (Table 3). Finally, we added together the contribution of each product to build the LR Score. Next, a Cox proportional hazards regression was carried out with the LR Score created in both the Training and the Validation Sets. The results showed an *unadjusted* hazard ratio (HR) of 2.72, and an *adjusted* (by age, gender, diet, BMI, treatment with statins, HDL-c, and TGs plasma levels) HR of 2.87 in the Training set. Meanwhile, in the Validation set, there was an *unadjusted* HR of 1.54, and an *adjusted* HR of 1.43, per one SD increase.

Next, the prediction power of the score created was evaluated by categorizing patients according to the LR Score by ascending tertiles, quartiles, and the median, in both the Training and the Validation set.

**Fig. 3:**
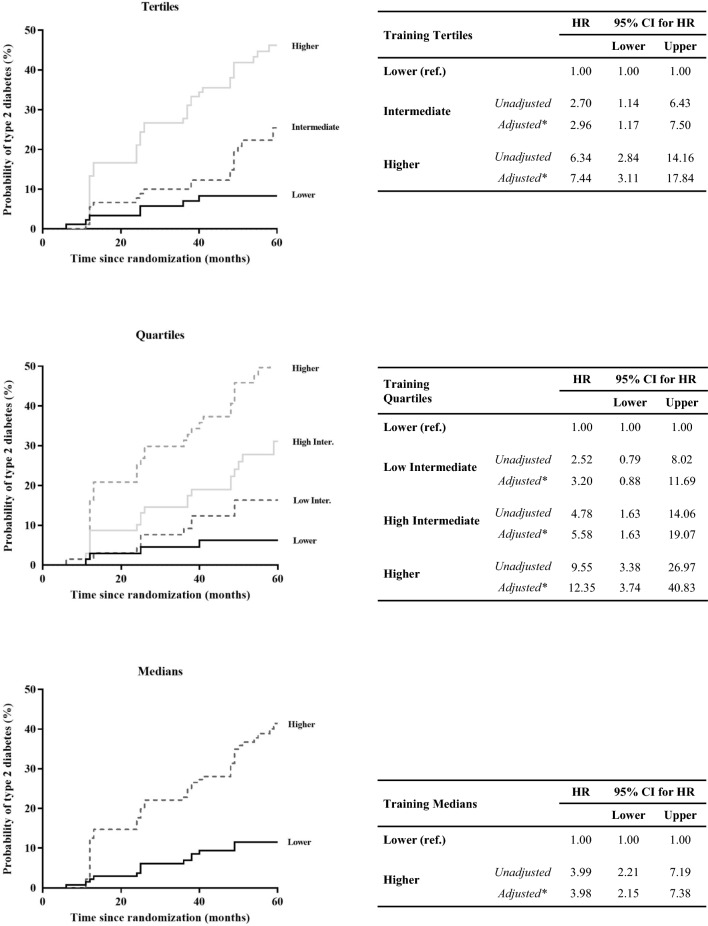
Disease-free survival by Cox proportional hazards regression analysis according to lipid species score in the Training Set. Patients from the Training set were categorized according to the Lipidomic Risk score by tertiles, quartiles, and median (in ascending order). *This model was adjusted for age, gender, diet, body mass index, high density lipoproteins-cholesterol, plasma triacylglycerols, and statin intensity treatment. The hazard ratio (HR) between groups was calculated. CI: confidence interval

In the Training set (Fig. 3), subjects from the High (*unadjusted* HR: 6.34; and *adjusted* HR: 7.44) and Intermediate (*unadjusted* HR: 2.70; and *adjusted* HR: 2.96) groups of the tertile classification showed significantly higher T2DM risk than the Low LR Score group. Besides, when the patients were classified into quartiles, subjects in the High (*unadjusted* HR: 9.55; and *adjusted* HR: 12.35) and the High-Intermediate (*unadjusted* HR: 4.78; and *adjusted* HR: 5.58) LR Score groups presented a greater risk of T2DM development when compared with the Low LR Score group (reference). Finally, when patients were classified by the median, a higher T2DM risk for patients in the High group (*unadjusted* HR: 3.99; and *adjusted* HR: 3.98) was observed compared with the Low LR Score group used as the reference.

**Fig. 4:**
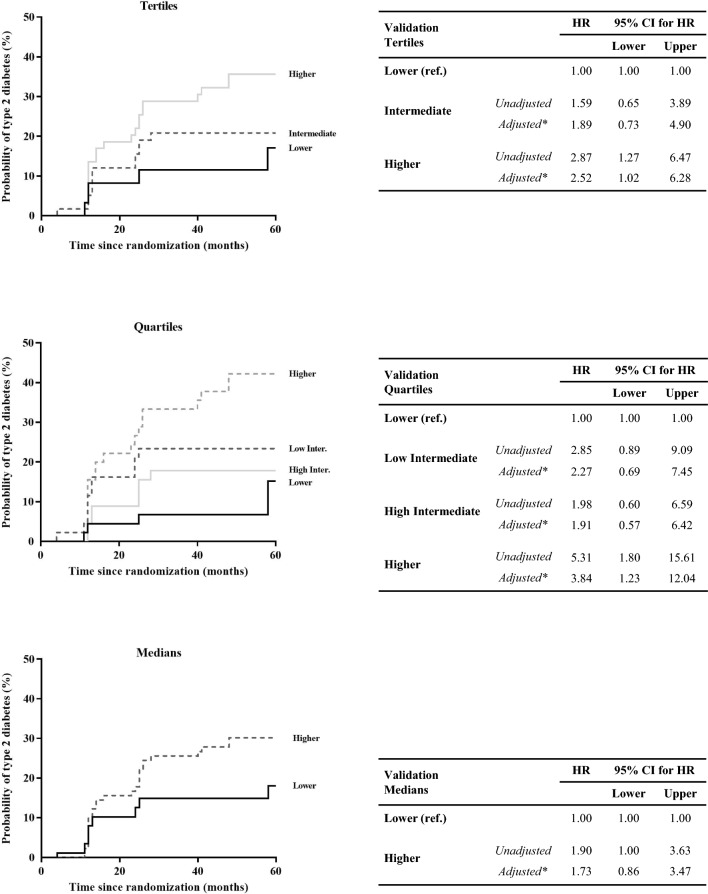
Disease-free survival by Cox proportional hazards regression analysis according to lipid species score in the Validation Set. Patients from the validation set were categorized according to the Lipidomic Risk score by tertiles, quartiles, and median (in ascending order). *This model was adjusted for age, gender, diet, body mass index, high density lipoproteins-cholesterol, plasma triacylglycerols, and statin intensity treatment. The hazard ratio (HR) between groups was calculated. CI: confidence interval

We also analysed the LR Score in the validation set (Fig. 4). When patients were categorized by tertiles, subjects in the High LR Score group (*unadjusted* HR: 2.87; and *adjusted* HR: 2.52) presented a higher T2DM risk than the group of patients classified in the Low LR Score group (reference). Moreover, when patients were organized by quartiles, we observed a higher T2DM risk in the High LR Score group (*unadjusted* HR: 5.31; and *adjusted* HR: 3.84), with the Low LR Score group as a reference. Finally, when patients were classified by the median, patients in the High group (*unadjusted* HR: 1.90; and *adjusted* HR: 1.73) had a higher risk of T2DM development compared to the Low LR Score group.

### Relationship between Lipidomic Risk score and insulin resistance and beta-cell functionality indexes

**Fig. 5:**
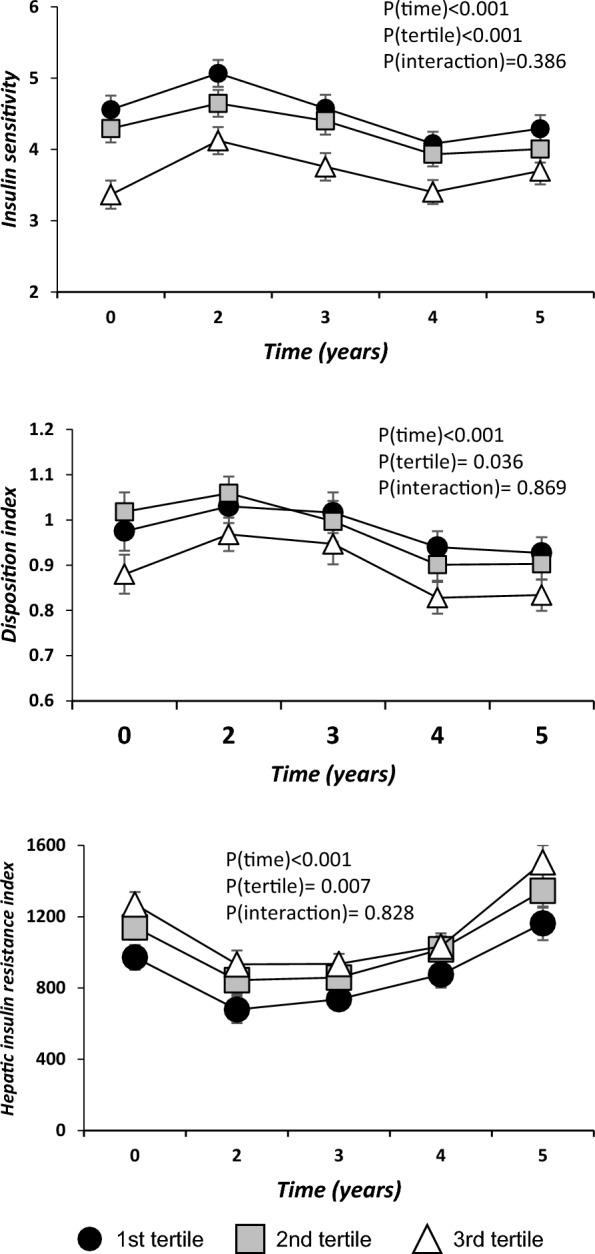
Relationship between lipid profile and insulin resistance and beta-cell functionality indexes according to the ascending tertiles of the Lipidomic Risk score. Patients were categorized according to the Lipidomic Risk score by ascending tertiles. ANOVA for repeated measures p-values adjusted by age, gender, diet, body mass index, high density lipoproteins-cholesterol, and plasma triacylglycerols. Global p-values: P(time): time effect; P(tertile): tertile of the Lipidomic Risk score effect; P(interaction): time by tertiles of the Lipidomic Risk score interaction

We also studied the relationship between LR Score, insulin resistance, and beta-cell function as assessed by validated indexes during the follow-up (Fig. 5). The patients were organized by ascending tertiles of the LR Score. It was found that subjects in the High LR Score group presented lower values of ISI than subjects with Low and Intermediate LR Scores (*P* < *0.001 and* = *P* = *0.011*, respectively). We also found that the High LR Score group presented lower values of DI than subjects with Low and Intermediate LR Scores (statistical trend; *P* = *0.070 and* = *P* = *0.081*, respectively). Moreover, the High LR Score group also presented higher values of the Hepatic insulin resistance index (HIRI) (*P* = *0.005*) compared with patients in the Low LR Score group.

## Discussion

Despite the determining role of dyslipidaemia in T2DM, the molecular mechanisms and the involvement of the specific lipid species behind this role are not yet well understood [21]. Over the last few years, lipidomics has been proposed as a method to elucidate the changes that occur in metabolism thanks to its precision in distinguishing between different lipids species [5]. In our study, we identified 15 lipid species, selected by RSF from a total of 440. These compounds were included in a lipid species-based score which was statistically associated with T2DM development risk. Moreover, patients with higher LR Score values have higher T2DM risk, lower insulin sensitivity as determined by the ISI index, and higher hepatic insulin resistance, as determined by the HIRI index.

T2DM is currently the most prevalent form of diabetes, affecting around 380 million people worldwide, and accounting for 90% of all cases. It is also on the rise, mainly due to the prevalence of sedentary lifestyles and inadequate diets [22]. Changes in lifestyle, including dietary interventions and exercise, have proven effective in preventing diabetes [23]; however, it remains difficult to predict which individuals will benefit from such interventions. This is particularly relevant in the case of patients with CHD, given that the co-occurrence of CHD together with T2DM significantly boosts the risk of macrovascular complications and mortality, and leads to around 80% of all deaths [4].

Current predictive models in T2DM research combine classic biomarkers and risk factors, including serum parameters, anthropometric characteristics, and factors related to lifestyle. On the FINDRISC questionnaire [24], the patient provides information on whether they have ever had high blood glucose levels and if they regularly take treatment for hypertension, along with information on age, gender, nutritional habits, and family history. On the other hand, the ADA questionnaire only includes information provided by the patient regarding age, gender, weight, family history, and physical activity. The predictive power of these models is moderate, and they include information provided by the patient, which reduces the reliability of the prediction of T2DM. Therefore, reliable, highly accurate predictive biomarkers are currently required to efficiently assess the risk of developing T2DM in clinical practice, which is especially important in patients with cardiovascular disease (CVD).

This study showed that the predictive capacity of the clinical variables was significantly improved by the addition of 15 lipid species, selected by RSF from a total of 440 determined by our experimental approach. To the best of our knowledge, this is the first time that a lipidomic study has been carried out in a risk population of CHD patients to predict diabetes incidence. Nevertheless, a previous study in a non-CVD population also observed an improvement in the prediction capacity of their model when lipids were added to conventional risk factors [8]. However, in our study, we observed an AUC increase from 64 to 81% with a CHD population, while only a small increase from 83 to 84% was obtained in the non-CVD population. This difference could be due to the sensibility and specificity of the lipidomic analytical technique employed [9]. Apart from this, which may have partially contributed to the predictive power of the model, we also need to take into account that dyslipidemia associated with the CVD population [25] may contribute to the greater statistical improvement in our model in comparison with the study carried out in the non-CVD population.

Unlike the study by Razquin et al., which described a lipid profile based only on lipid classes associated with T2DM incidence, our study shows that we need to analyse individual lipid species to accurately differentiate the directionality of the association with T2DM. We identified four members of the PE lipid family, of which two, PE(16:0_18:1), and PE(O-20:0/18:0), were associated with the development of T2DM, whereas the other two, PE(16:1_18:1) and PE(18:0_18:2), were protective against T2DM. Moreover, while the relationship of PE with T2DM risk has been previously reported, the specific species and isomers have not been described previously [8]. Currently, there is little to be found in the literature on the role of compounds at this level of detail [26], suggesting that advances need to be made in methodology and defining lipid isomerism if we are to finally understand the mechanism of dyslipidaemia which occurs during the development of T2DM. Overall, PEs are involved in the mechanisms modifying membrane characteristics and the functionality of the transporters, receptors, channels, and enzymes. Here, our results suggest that the abundance of specific species and isomers and the proportions shown between them could differentially modulate the membrane characteristics promoting or protecting against diabetes, in turn affecting the functionality of insulin receptors and/or glucose transporter [27]. This idea is supported by the fact that the isomerism of other compounds (namely branched fatty acids esters of hydroxy fatty acids) has previously been proven to be associated with diabetes, which highlights the relevance of isomerism in this biological function [28].

Similarly, two compounds from the PC family were identified by the RSF as associated with T2DM in opposite ways, one protecting and another promoting the disease. PC(P-16:0/18:1) is linked with a protective role against the disease, while PC(P-16:1/18:0) is associated with diabetes development. PC is the only phospholipid essential for the assembly, secretion, and regulation of lipoproteins such as LDL and HDL [29]. Indeed, we tested the potential relationship between the levels of these PCs with LDL and HDL. We found a positive correlation between PC(P-16:0/18:1) and HDL plasma levels (data not shown). This finding supports the hypothesis of the protective role of PC(P-16:0/18:1) against T2DM development. The mechanism behind this process could be based on reduced HDL cholesterol levels associated with T2DM [30]. Although PC is required for the proper functioning of the metabolism, unusually high concentrations have been previously reported in cases of insulin resistance, T2DM, and metabolic syndrome [31, 32]. It has also been previously reported that PC is the mediator in decreasing insulin sensitivity in mice when high-fat diets are consumed [33–35]. However, scarce literature reports the role of PCs to this level of detail, complicating the understanding of the role in T2DM.

In contrast, isomers identified within the TGs, phosphatidylserines (PSs), and LPC are unidirectional. Among these three families, LPC is the only one with a protective role in preventing diabetes development. LPC is a hydrolysis product derived from the catalysis of phosphatidylcholine by phospholipase A_2_. Previous studies have linked the bidirectional role of LPCs with the regulation of glucose metabolism [32, 36], and experiments in lipidomics have identified that LPC(20:1) decreases in high-fat diets in mouse models, due to its association with HOMA-IR [5]. This study also identified that LPC (20:1) was negatively associated with BMI in humans and plasma insulin levels. Also, other LPCs different from LPC(20:1), such as LPC(18:2), were associated with a higher risk of developing glucose intolerance. Therefore, LPC(18:2) was suggested as a potential predictor for T2DM development [37]. Research into the role of LPC has identified that it activates the uptake of glucose by the adipocytes mediated by the GLUT4, consequently lowering the levels of glucose in the blood in murine models of diabetes [38, 39]. Consequently, we suspect that modulations in LPC (20:1) may play a role in developing systemic insulin resistance. In conclusion, unravelling the role of LPC in glucose homeostasis and insulin resistance mechanisms may contribute to increasing our knowledge of the mechanisms behind T2DM development.

Finally, it is important to mention the limitations of this study. Firstly, this research is based on a long-term, closely controlled dietary intervention, which, despite ensuring the quality of the study, may not reflect the level of compliance in a free-living population.

The second limitation is that the incidence of T2DM was not the primary endpoint of the CORDIOPREV trial, although it was a secondary objective of this study. Indeed, our study has the limitation that the incident-DIAB group has higher baseline glucose levels and an unbalanced number of men and women included as participants. In fact, this population was included in the CORDIOPREV study without any type of selection, therefore representing the sexual dimorphism existent in CHD, and any attempt to balance the number of men and women may introduce a bias. Moreover, the study included patients with coronary heart disease, which limits our findings to people with these characteristics and precludes its generalization to healthy individuals. Although diabetes prediction is extremely important since patients with acute myocardial infarction and T2DM have a considerably higher risk of developing a new cardiovascular event than those without T2DM [40], validation in a cohort without cardiovascular disease and with a closer profile to the general population would allow us to apply these methods to the general population.

## Conclusion

Overall, this study has shown the potential of highly sensitive lipidomics in identifying patients at risk of developing T2DM. In addition, the lipid species identified as associated with T2DM development, combined with clinical variables, have provided a new, highly sensitive model to be used in clinical practice. The findings also suggest that the risk of T2DM development is associated with a specific lipidomic profile which is characterized by lower peripheral insulin sensitivity and higher hepatic insulin resistance. Finally, these results also indicate that we need to look closely at isomers to understand the role of this specific compound in T2DM development since isomers of the same class of lipids are associated with different outcomes.

### Supplementary Information

**Additional file 1: Table S1.** Baseline medication of the type 2 diabetes mellitus incidence study. Data are n (%).

**Additional file 2: Table S2. **Characteristics of the population for type 2 diabetes mellitus incidence study after a median follow-up of 60 months. The ongoing patients for type 2 diabetes mellitus incidence study after a median follow-up of 60 months were 438 (107 in Incident-DIAB and 331 in Non-DIAB group). Means values ± S.E.M. Incident-DIAB: patients who developed T2DM but were non-diabetic at baseline. Non-DIAB: non-diabetic patients. BMI: body mass index. HbA1c: glycated hemoglobin A1c. ISI: insulin sensitivity index. IGI: insulinogenic index. One-way ANOVA P-values.

## Data Availability

Collaborations with the Cordioprev Study are open to Biomedical Institutions, always after an accepted proposal for scientific work. Depending on the nature of the collaboration, electronic data, hard copy data, or biological samples should be provided. All collaborations will be made after a collaboration agreement. Terms of the collaboration agreement will be specific for each collaboration, and the extent of the shared documentation (ie, deidentified participant data, data dictionary, biological samples, hard copy, or other specified data sets) will be also specifically set on the light of each work.

## Abbreviations

ADA: American Diabetes Association
ANOVA: Analysis of variance
AUC: Area under the curve
BMI: Body mass index
CEs: Cholesterol esters
CHD: Coronary heart disease
CVD: Cardiovascular disease
DAGs: Diacylglycerols
HDL: High-density lipoproteins
HR: Hazard ratio
Incident-DIAB: Non-diabetic patients at baseline who developed T2DM after a median follow-up of 60 months
ISI: Insulin sensitivity index
IGI: Insulinogenic index
DI: Disposition index
LC: Liquid chromatography
LDL: Low-density lipoproteins
LPCs: Lysophosphatidylcholines
LR: Lipidomic Risk
MS: Mass spectrometry
MUFA: Monounsaturated fatty acids
non-DIAB: Non-diabetic patients at baseline who did not develop T2DM after a median follow-up of 60 months
OGTT: Oral glucose tolerance test
PC-PLs: Phosphatidylcholine-plasmalogens
PCs: Lysophosphatidylcholines
PEs: Phosphatidylethanolamines
PG: Phosphatidyl glycerol
PI: Phosphatidyl inositol
PSs: Phosphatidylserines
PUFA: Poly-unsaturated fatty acids
ROC: Receiver operating characteristic
RSF: Random Survival Forest
SD: Standard deviation
SEM: Standard error of the mean
SMs: Sphingomyelins
T2DM: Type 2 diabetes mellitus
TGs: Triacylglycerols

## References

[1] Strain WD, Paldanius PM (2018). Diabetes, cardiovascular disease and the microcirculation. Cardiovasc Diabetol.

[2] Vilas-Boas EA, Almeida DC, Roma LP, Ortis F, Carpinelli AR (2021). Lipotoxicity and beta-cell failure in type 2 diabetes: oxidative stress linked to NADPH oxidase and ER stress. Cells..

[3] Ogurtsova K, da Rocha Fernandes JD, Huang Y, Linnenkamp U, Guariguata L, Cho NH, Cavan D, Shaw JE, Makaroff LE (2017). IDF diabetes atlas: global estimates for the prevalence of diabetes for 2015 and 2040. Diabetes Res Clin Pract.

[4] DeFronzo RA (2010). Insulin resistance, lipotoxicity, type 2 diabetes and atherosclerosis: the missing links. The Claude Bernard Lecture 2009. Diabetologia..

[5] Barber MN, Risis S, Yang C, Meikle PJ, Staples M, Febbraio MA, Bruce CR (2012). Plasma lysophosphatidylcholine levels are reduced in obesity and type 2 diabetes. PLoS ONE.

[6] Pirillo A, Casula M, Olmastroni E, Norata GD, Catapano AL (2021). Global epidemiology of dyslipidaemias. Nat Rev Cardiol.

[7] Tomlinson B, Patil NG, Fok M, Lam CWK (2021). Managing dyslipidemia in patients with type 2 diabetes. Expert Opin Pharmacother.

[8] Razquin C, Toledo E, Clish CB, Ruiz-Canela M, Dennis C, Corella D, Papandreou C, Ros E, Estruch R, Guasch-Ferre M (2018). Plasma lipidomic profiling and risk of type 2 diabetes in the PREDIMED Trial. Diabetes Care.

[9] Lopez-Bascon MA, Calderon-Santiago M, Diaz-Lozano A, Camargo A, Lopez-Miranda J, Priego-Capote F (2020). Development of a qualitative/quantitative strategy for comprehensive determination of polar lipids by LC-MS/MS in human plasma. Anal Bioanal Chem.

[10] Delgado-Lista J, Perez-Martinez P, Garcia-Rios A, Alcala-Diaz JF, Perez-Caballero AI, Gomez-Delgado F, Fuentes F, Quintana-Navarro G, Lopez-Segura F, Ortiz-Morales AM (2016). CORonary Diet Intervention with Olive oil and cardiovascular PREVention study (the CORDIOPREV study): rationale, methods, and baseline characteristics—a clinical trial comparing the efficacy of a Mediterranean diet rich in olive oil versus a low-fat diet on cardiovascular disease in coronary patients. Am Heart J.

[11] Association AD (2019). 2. Classification and Diagnosis Of Diabetes: Standards Of Medical Care In Diabetes-2019. Diabetes Care..

[12] Quintana-Navarro GM, Alcala-Diaz JF, Lopez-Moreno J, Perez-Corral I, Leon-Acuna A, Torres-Pena JD, Rangel-Zuniga OA, Arenas de Larriva AP, Corina A, Camargo A (2019). Long-term dietary adherence and changes in dietary intake in coronary patients after intervention with a Mediterranean diet or a low-fat diet: the CORDIOPREV randomized trial. Eur J Nutr.

[13] Fernandez-Ballart JD, Pinol JL, Zazpe I, Corella D, Carrasco P, Toledo E, Perez-Bauer M, Martinez-Gonzalez MA, Salas-Salvado J, Martin-Moreno JM (2010). Relative validity of a semi-quantitative food-frequency questionnaire in an elderly Mediterranean population of Spain. Br J Nutr.

[14] Martinez-Gonzalez MA, Fernandez-Jarne E, Serrano-Martinez M, Wright M, Gomez-Gracia E (2004). Development of a short dietary intake questionnaire for the quantitative estimation of adherence to a cardioprotective Mediterranean diet. Eur J Clin Nutr.

[15] Blanco-Rojo R, Alcala-Diaz JF, Wopereis S, Perez-Martinez P, Quintana-Navarro GM, Marin C, Ordovas JM, van Ommen B, Perez-Jimenez F, Delgado-Lista J (2016). The insulin resistance phenotype (muscle or liver) interacts with the type of diet to determine changes in disposition index after 2 years of intervention: the CORDIOPREV-DIAB randomised clinical trial. Diabetologia.

[16] Hsu FF, Turk J (2002). Characterization of ceramides by low energy collisional-activated dissociation tandem mass spectrometry with negative-ion electrospray ionization. J Am Soc Mass Spectrom.

[17] Ishwaran H, Kogalur UB (2010). Consistency of random survival forests. Stat Probab Lett.

[18] Dietrich S, Floegel A, Troll M, Kühn T, Rathmann W, Peters A, Sookthai D, von Bergen M, Kaaks R, Adamski J (2016). Random Survival Forest in practice: a method for modelling complex metabolomics data in time to event analysis. Int J Epidemiol.

[19] Kuhn M (2008). Building predictive models in R using the caret package. J Stat Softw.

[20] Robin X, Turck N, Hainard A, Tiberti N, Lisacek F, Sanchez JC, Muller M (2011). pROC: an open-source package for R and S+ to analyze and compare ROC curves. BMC Bioinformatics.

[21] Kane JP, Pullinger CR, Goldfine ID, Malloy MJ (2021). Dyslipidemia and diabetes mellitus: role of lipoprotein species and interrelated pathways of lipid metabolism in diabetes mellitus. Curr Opin Pharmacol.

[22] Diabetes. Who.int s/f [http://www.who.int/diabetes/en/]. Accessed 7 Feb 2023.

[23] Uusitupa M, Khan TA, Viguiliouk E, Kahleova H, Rivellese AA, Hermansen K, Pfeiffer A, Thanopoulou A, Salas-Salvado J, Schwab U (2019). Prevention of type 2 diabetes by lifestyle changes: a systematic review and meta-analysis. Nutrients..

[24] Lindstrom J, Tuomilehto J (2003). The diabetes risk score: a practical tool to predict type 2 diabetes risk. Diabetes Care.

[25] Wong ND (2014). Epidemiological studies of CHD and the evolution of preventive cardiology. Nat Rev Cardiol.

[26] Eichelmann F, Sellem L, Wittenbecher C, Jager S, Kuxhaus O, Prada M, Cuadrat R, Jackson KG, Lovegrove JA, Schulze MB (2022). Deep lipidomics in human plasma: cardiometabolic disease risk and effect of dietary fat modulation. Circulation.

[27] Lopez S, Bermudez B, Abia R, Muriana FJ (2010). The influence of major dietary fatty acids on insulin secretion and action. Curr Opin Lipidol.

[28] Aryal P, Syed I, Lee J, Patel R, Nelson AT, Siegel D, Saghatelian A, Kahn BB (2021). Distinct biological activities of isomers from several families of branched fatty acid esters of hydroxy fatty acids (FAHFAs). J Lipid Res.

[29] Lent-Schochet D, McLaughlin M, Ramakrishnan N, Jialal I (2019). Exploratory metabolomics of metabolic syndrome: a status report. World J Diabetes.

[30] Xepapadaki E, Nikdima I, Sagiadinou EC, Zvintzou E, Kypreos KE (2021). HDL and type 2 diabetes: the chicken or the egg?. Diabetologia.

[31] Fiehn O, Garvey WT, Newman JW, Lok KH, Hoppel CL, Adams SH (2010). Plasma metabolomic profiles reflective of glucose homeostasis in non-diabetic and type 2 diabetic obese African-American women. PLoS ONE.

[32] Liu P, Zhu W, Chen C, Yan B, Zhu L, Chen X, Peng C (2020). The mechanisms of lysophosphatidylcholine in the development of diseases. Life Sci.

[33] Kumar A, Sundaram K, Mu J, Dryden GW, Sriwastva MK, Lei C, Zhang L, Qiu X, Xu F, Yan J (2021). High-fat diet-induced upregulation of exosomal phosphatidylcholine contributes to insulin resistance. Nat Commun.

[34] van der Veen JN, Kennelly JP, Wan S, Vance JE, Vance DE, Jacobs RL (2017). The critical role of phosphatidylcholine and phosphatidylethanolamine metabolism in health and disease. Biochim Biophys Acta Biomembr..

[35] Walker AK, Jacobs RL, Watts JL, Rottiers V, Jiang K, Finnegan DM, Shioda T, Hansen M, Yang F, Niebergall LJ (2011). A conserved SREBP-1/phosphatidylcholine feedback circuit regulates lipogenesis in metazoans. Cell.

[36] Han MS, Lim YM, Quan W, Kim JR, Chung KW, Kang M, Kim S, Park SY, Han JS, Park SY (2011). Lysophosphatidylcholine as an effector of fatty acid-induced insulin resistance. J Lipid Res.

[37] Wang-Sattler R, Yu Z, Herder C, Messias AC, Floegel A, He Y, Heim K, Campillos M, Holzapfel C, Thorand B (2012). Novel biomarkers for pre-diabetes identified by metabolomics. Mol Syst Biol.

[38] Huynh K, Barlow CK, Jayawardana KS, Weir JM, Mellett NA, Cinel M, Magliano DJ, Shaw JE, Drew BG, Meikle PJ (2019). High-throughput plasma lipidomics: detailed mapping of the associations with cardiometabolic risk factors. Cell Chem Biol..

[39] Yea K, Kim J, Yoon JH, Kwon T, Kim JH, Lee BD, Lee HJ, Lee SJ, Kim JI, Lee TG (2009). Lysophosphatidylcholine activates adipocyte glucose uptake and lowers blood glucose levels in murine models of diabetes. J Biol Chem.

[40] Martin-Timon I, Sevillano-Collantes C, Segura-Galindo A, Del Canizo-Gomez FJ (2014). Type 2 diabetes and cardiovascular disease: Have all risk factors the same strength?. World J Diabetes.

