# Supplementary material for: Plasma lipidic fingerprint associated with type 2 diabetes in patients with coronary heart disease: CORDIOPREV study
Source: Cardiovasc Diabetol. 2023 Aug 3;22:199. doi: 10.1186/s12933-023-01933-1 (PMC10401778; doi:10.1186/s12933-023-01933-1)
Supplement: Supplementary file 1 — Additional file 1: Table S1. Baseline medication of the type 2 diabetes mellitus incidence study. Data are n (%). [file 12933_2023_1933_MOESM1_ESM.docx]

| ***Baseline medication*** | ***All patients*** | ***Incident-DIAB*** | ***Non-DIAB*** |
| --- | --- | --- | --- |
| *Antiplatelets or anticoagulants* | 443 (95.9%) | 100 (93.5%) | 343 (96.6%) |
| *Statins* | 393 (85.1%) | 88 (82.2%) | 305 (85.9%) |
| *Other lipid-lowering drugs* | 104 (22.5%) | 30 (28.0%) | 74 (20.8%) |
| *Angiotensin-converting enzyme inhibitors or angiotensin II receptor blockers* | 368 (79.7%) | 85 (79.4%) | 283 (79.7%) |
| *β-blockers* | 372 (80.5%) | 87 (81.3%) | 285 (80.3%) |
| *Calcium antagonist* | 101 (21.9%) | 29 (27.1%) | 72 (20.3%) |
| *Diuretics* | 190 (41.1%) | 57 (53.3%) | 133 (37.5%) |

**Table S1. Baseline medication of the type 2 diabetes mellitus incidence study.** Data are n (%).
