# Supplementary material for: Plasma lipidic fingerprint associated with type 2 diabetes in patients with coronary heart disease: CORDIOPREV study
Source: Cardiovasc Diabetol. 2023 Aug 3;22:199. doi: 10.1186/s12933-023-01933-1 (PMC10401778; doi:10.1186/s12933-023-01933-1)
Supplement: Supplementary file 2 — Additional file 2: Table S2. Characteristics of the population for type 2 diabetes mellitus incidence study after a median follow-up of 60 months. The ongoing patients for type 2 diabetes mellitus incidence study after a median follow-up of 60 months were 438 (107 in Incident-DIAB and 331 in Non-DIAB group). Means values ± S.E.M. Incident-DIAB: patients who developed T2DM but were non-diabetic at baseline. Non-DIAB: non-diabetic patients. BMI: body mass index. HbA1c: glycated hemoglobin A1c. ISI: insulin sensitivity index. IGI: insulinogenic index. One-way ANOVA P-values. [file 12933_2023_1933_MOESM2_ESM.docx]

|  | Incident-DIAB | Non-DIAB | P-value |
| --- | --- | --- | --- |
| *Weight (kg)* | 85.44±1.50 | 82.65±0.78 | 0.084 |
| *BMI (kg/m^2^)* | 31.37±0.49 | 29.91±0.25 | 0.005 |
| *Waist circumference (cm)* | 106.96±1.14 | 102.11±0.61 | <0.001 |
| *Serum triacylglycerols (mg/dL)* | 135.12±7.14 | 110.10±2.83 | <0.001 |
| *Total cholesterol (mg/dL)* | 160.76±3.34 | 153.43±1.52 | 0.026 |
| *HDL-cholesterol (mg/dL)* | 43.18±0.99 | 43.63±0.53 | 0.682 |
| *LDL-cholesterol (mg/dL)* | 90.54±2.79 | 87.28±1.26 | 0.233 |
| *CRP (mg/L)* | 3.04±0.30 | 2.25±0.18 | 0.031 |
| *HbA1c ( %)* | 5.81±0.04 | 5.61±0.02 | <0.001 |
| *Fasting Glucose (mg/dL)* | 102.61±1.30 | 93.60±0.53 | <0.001 |
| *Fasting Insulin (mU/L)* | 13.27±1.10 | 9.65±0.30 | <0.001 |
| *ISI* | 3.12±0.19 | 4.28±0.14 | <0.001 |
| *HOMA-IR* | 4.16±0.33 | 3.03±0.15 | <0.001 |
| *IGI* | 0.95±0.16 | 0.88±0.07 | 0.642 |
| *Disposition Index* | 0.67±0.03 | 0.96±0.03 | <0.001 |

**Table S2.** Characteristics of the population for type 2 diabetes mellitus incidence study after a median follow-up of 60 months. The ongoing patients for type 2 diabetes mellitus incidence study after a median follow-up of 60 months were 438 (107 in Incident-DIAB and 331 in Non-DIAB group). Means values ± S.E.M. Incident-DIAB: patients who developed T2DM but were non-diabetic at baseline. Non-DIAB: non-diabetic patients. BMI: body mass index. HbA1c: glycated hemoglobin A1c. ISI: insulin sensitivity index. IGI: insulinogenic index. One-way ANOVA P-values.
